# A machine learning approach on multiscale texture analysis for breast microcalcification diagnosis

**DOI:** 10.1186/s12859-020-3358-4

**Published:** 2020-03-11

**Authors:** Annarita Fanizzi, Teresa M. A. Basile, Liliana Losurdo, Roberto Bellotti, Ubaldo Bottigli, Rosalba Dentamaro, Vittorio Didonna, Alfonso Fausto, Raffaella Massafra, Marco Moschetta, Ondina Popescu, Pasquale Tamborra, Sabina Tangaro, Daniele La Forgia

**Affiliations:** 1 I.R.C.C.S. Istituto Tumori “Giovanni Paolo II”, viale O. Flacco 65, Bari, Italy; 2 Dip. Interateneo di Fisica “M. Merlin”, Università degli Studi di Bari “A. Moro”, via G. Amendola 173, Bari, Italy; 3 INFN - Istituto Nazionale di Fisica Nucleare, sezione di Bari, via G. Amendola 173, Bari, Italy; 4 Dip. di Scienze Fisiche, della Terra e dell’Ambiente, Università degli Studi di Siena, strada Laterina 2, Siena, Italy; 5 Dip. di Diagnostica delle Immagini, Ospedale Universitario di Siena, viale Bracci 16, Siena, Italy; 6 Dip. Interdisciplinare di Medicina, Università degli Studi di Bari “A. Moro”, piazza G. Cesare 11, Bari, Italy

**Keywords:** Computer-aided diagnosis, Microcalcifications, Digital mammograms, Haar wavelet transform, SURF, Minimum eigenvalue algorithm, Random forest, Feature selection

## Abstract

**Background:**

Screening programs use mammography as primary diagnostic tool for detecting breast cancer at an early stage. The diagnosis of some lesions, such as microcalcifications, is still difficult today for radiologists. In this paper, we proposed an automatic binary model for discriminating tissue in digital mammograms, as support tool for the radiologists. In particular, we compared the contribution of different methods on the feature selection process in terms of the learning performances and selected features.

**Results:**

For each ROI, we extracted textural features on Haar wavelet decompositions and also interest points and corners detected by using Speeded Up Robust Feature (SURF) and Minimum Eigenvalue Algorithm (MinEigenAlg). Then a Random Forest binary classifier is trained on a subset of a sub-set features selected by two different kinds of feature selection techniques, such as filter and embedded methods. We tested the proposed model on 260 ROIs extracted from digital mammograms of the BCDR public database. The best prediction performance for the normal/abnormal and benign/malignant problems reaches a median AUC value of 98.16*%* and 92.08*%*, and an accuracy of 97.31*%* and 88.46*%*, respectively. The experimental result was comparable with related work performance.

**Conclusions:**

The best performing result obtained with embedded method is more parsimonious than the filter one. The SURF and MinEigen algorithms provide a strong informative content useful for the characterization of microcalcification clusters.

## Background

Breast cancer is the first cause of death among women and, although it is difficult to prevent, an early diagnosis of breast lesions increases the chances of survival and reduce the mortality rate [1]. Currently, screening programs use mammography [2, 3] as primary diagnostic tool for detecting breast cancer at an early stage. However the identification of some lesions remains still difficult for radiologists. In particular, the 55% of breast diseases with tumor lesions are accompanied by the presence of microcalcifications (MCs), that are tiny spots of calcium deposits localized or broadly diffused on the breast areas, especially when they appear extremely minute (sometimes they do not exceed 0.1 mm) and grouped in clusters.

The diagnosis of microcalcifications is usually based on radiologists expertise resulting in some cases in inaccurate lesion detection [4–6] or in performing unnecessary breast biopsies on benign calcification clusters. This limit becomes more evident in women with dense breast tissue that can hide lesions causing cancer to be detected at later stages [7, 8]. To overcome such limits, a solution is represented by the double blind reading of the mammograms by two radiologists [9] with a consequent higher workload and cost. A more interesting solution could be represented by using intelligent techniques to automatize the process of identification, normal vs abnormal tissue, and diagnosis, benign vs malignant, of clustered microcalcifications.

Several works have presented computerized methods to detect abnormalities in mammograms, playing a key role in the early detection of breast cancer thus helping to reduce the mortality rate due to breast pathologies in a cost-effective manner [5]. Such methods are known as Computer-Aided Detection/Diagnosis (CAD) systems and may offer to radiologists a reliable support in the evaluation of mammographic images [4, 10–12].

Many methods have been proposed to achieve a robust mammography-based CAD system for microcalcification diagnosis [13–18], in some cases well performing in dealing with specific abnormalities. Nevertheless, the automatic and accurate classification of microcalcification clusters, especially in differentiating the benign from the malignant ones, remains still complicated due to their nature. For this reason, the focus of this study is on the identification of a general model able to discriminate and, at the same time, characterize breast tissue and lesions with the aim of facing the fundamental challenge of improving the accuracy of breast lesion identification in order to decrease unnecessary biopsies and later surgeries. Accordingly, in the proposed model, an important role should be played by the features extraction and selection process, used to describe and characterize the regions of interest (ROIs), as well as by the classifier employed in the last phase of the CAD scheme that should be responsible of the decision regarding the origin, benign or malignant, of the region.

For what concerns the extraction of a representative set of features from the ROIs, different models have been proposed in literature ranging from those describing shapes of the clusters and classical statistical measurements [10, 19] to the ones exploiting morphological features [5, 16]. In some works, the texture analysis model uses a set of local statistical properties of pixel intensity. The textural features are obtained by the decomposition of the image into different frequency sub-bands by a wavelet transform [13, 14, 20] or by considering the spatial relationship between pixels with different gray-levels using the gray level co-occurrence-matrix [21–24]. Differently from textural descriptions of the breast lesions, some works concentrate on the potential correlation between the topology of clustered MCs and their pathological nature [17, 25]. Overall, all the works reported in literature use a broad variety of machine learning techniques such as k-Nearest Neighbours (kNN) [17, 23], Artificial Neural Networks (ANN) [14], and Support Vector Machines (SVM) [13, 14, 21] to build a classifier model able to discriminate the ROIs containing microcalcifications as benign or malignant using the extracted feature sets.

The development and integration of such a tool able to classify breast regions seem to be the natural prosecution of work presented in [26] where a CAD system working on full-field digital mammograms for the detection of clustered microcalcifications is reported. Indeed, as a succeeding step one expects the CAD system to perform a classification of the identified regions firstly in normal or abnormal tissue, as to reduce the erroneously detected regions that represent the false positive instances, and successively, on the abnormal classified regions, in benign or malignant lesions so to reduce recalls for unnecessary and stressful biopsies or ultrasound scans.

In this work, we propose the exploitation of texture analysis methods combined with machine learning techniques in order to characterize breast regions into normal/abnormal tissue and successively into benign/malignant lesions. The process is a multi-phase approach made up of a feature extraction step, performed by texture analysis methods, a features selection step, carried out with filter and embedded methods, and a breast region classification, performed by means of machine learning techniques, in order to categorize clusters of MCs in digital mammograms. Specifically, for each ROI a set of well-defined textural features, such standard statistical features, on a multiscale decomposition of the image based on the Haar wavelet transform [27, 28] are extracted. Moreover, interest points and corners are detected by using Speeded Up Robust Feature (SURF) [29] and Minimum Eigenvalue Algorithm (MinEigenAlg) [30], respectively. Successively, two different kinds of feature selection techniques, such as filter and embedded methods [31, 32] are exploited. Specifically, the filter methods include algorithms that evaluate the capacity of the individual features to predict the expected result. Embedded methods allow optimization between the interaction of the selected features and the classification algorithm used. Finally, a training test by means of a state of art classifier, such as Random Forest [33], is performed to classify the clustered microcalcifications. The proposed approach was tested on full-field digital mammograms extracted from the public database BCDR (Breast Cancer Digital Repository −https://bcdr.ceta-ciemat.es) [34]. The model performance was tested in cross-validation and evaluated in terms of accuracy, sensitivity and specificity, obtaining results in agreement with the literature.

## Materials and methods

### Dataset selection

The image dataset consisted of a set of digital mammograms randomly selected from the Breast Cancer Digital Repository [34] (BCDR). Currently, the BCDR contains cases of 1734 patients with mammography and ultrasound images, clinical history, lesion segmentation and selected pre-computed image-based descriptors. Patient cases are classified according to BIRADS categories [35] and annotated by specialized radiologists covering all the possibilities of diagnosis. In the database all available medio-lateral oblique (MLO) and cranial caudal (CC) views of the left and right breast are included. The BCDR is subdivided in two different repositories: (1) a Film Mammography-based Repository (BCDR-FM) and (2) a Full Field Digital Mammography-based Repository (BCDR-DM). In particular, BCDR-DM includes 724 patient cases with digital mammograms. The MLO and CC images are gray-level mammograms with a resolution of 3328 (width) by 4084 (height) or 2560 (width) by 3328 (height) pixels, depending on the compression plate used in the acquisition (according to the breast size of the patient).

For this study, digital mammograms from BCDR-DM both in MLO and CC views were considered. Since the BCDR images reported the segmentation of the main lesions only, each extracted image was evaluated in double blind by two radiologists of our Institute dedicated to senological diagnostics, which have manually identified and classified ROIs containing the microcalcification clusters. Then, after a comparison between these independent readings, only ROIs for which both radiologists agreed were taken into account. As result, the dataset exploited in this study consists of 130 ROIs with clustered MCs, where 75 benign and 55 malignant, and 130 ROIs without any pathology.

### Textural feature extraction

In this paper, we propose a fully automated model for the characterization of regions containing clustered microcalcifications in digital mammograms mainly based on a texture analysis approach. Since a fundamental property of the image texture is the scale at which the image is observed and analyzed, in this work a wavelet transform based on multiscale texture analysis approach, and specifically the Haar wavelet transform, was exploited. The Haar wavelet [27, 28] is a sequence of rescaled “square-shaped” functions which together form a wavelet family or basis. Wavelet approach is similar to Fourier analysis in that it allows a target function over an interval to be represented in terms of an orthonormal basis. This basis is composed by scaled and translated basis functions and denoted as *ϕ*(*x*,*y*) and *ψ*(*x*,*y*). Conceptually, the scaling function is the low frequency component of the scaling function in 2 dimensions, and therefore there is one 2D scaling function. The translated function has three different wavelet components, namely horizontal, vertical and diagonal. However, the wavelet function is related to the order at which apply the low- and high-filters and, since the wavelet function is separable, i.e.

$$f(x,y)=f_{1}(x)f_{2}(y)$$

these functions can be written as follows:

1 $$ \begin{aligned} \phi(x,y)& = \phi(x)\phi(y) \rightarrow LL=\mathrm{low-low}, \\ \psi^{H}(x,y)& = \psi(x)\phi(y) \rightarrow HL=\mathrm{high-low},\\ \psi^{V}(x,y)& = \phi(x)\psi(y) \rightarrow LH=\mathrm{low-high},\\ \psi^{D}(x,y)& = \psi(x)\psi(y) \rightarrow HH=\mathrm{high-high}, \end{aligned}   $$

where the corresponding filter order is denoted [27].

**Fig. 1:**
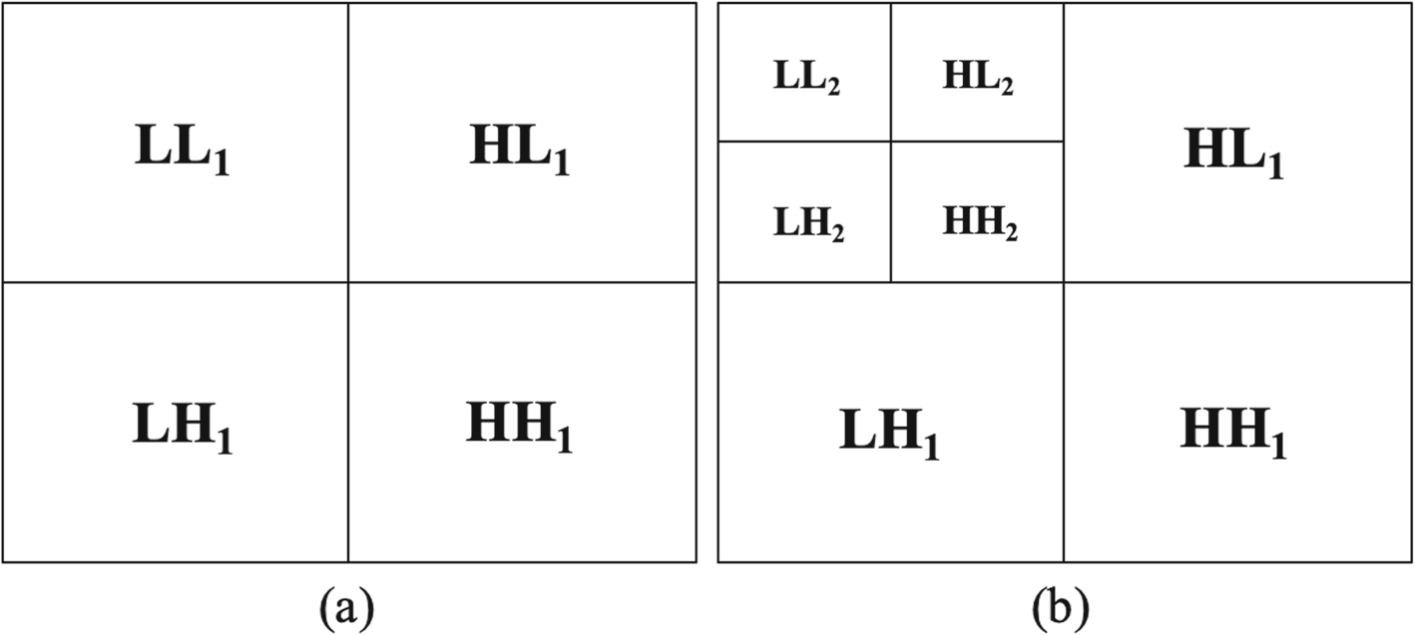
Image Haar decomposition. **a** One- and **b** two- level of decomposition

The Haar sequence is recognized as the first known wavelet basis and extensively used as a teaching example. In the 2D Haar wavelet decomposition on the image, the original image is first low-pass filtered and downscaled, yielding an approximation coefficients sub-image (*LL*_1_ in Fig. 1a, top left), and then high-pass filtered, yielding the three detail coefficients sub-images (Fig. 1a, top right: horizontal *HL*_1_, bottom left: vertical *LH*_1_, and bottom right: diagonal *HH*_1_), according to the general form of 2D wavelet transform (Eq. 1). To compute the successive level of decomposition, the process is iterated on *LL*_1_, i.e. the approximation coefficient sub-image (Fig. 1b, top left). Specifically, in this study, we performed the 2D Haar Transform at two levels of decomposition.

However, to perform texture analysis, a number of attributes or descriptors that differentiate the textures have to be identified. Of course, such descriptors are assumed to be uniform within the regions with the same texture. Many works in literature report the texture analysis process based on first- or second-order statistics computed on the image histogram. The use of such texture descriptors rely on the assumption that texture can be defined by local statistical properties of pixel gray levels. For this reason, in our study, for each of the eight sub-images obtained in the Haar decomposition (*LL*, *HL*, *LH* and *HH* for levels 1 and 2) the following features are computed: mean, variance, skewness, kurtosis, entropy, relative smoothness; thus resulting, for each ROI, in a set of 48 Statistical Features (SF set).

### Interest point/corner detection

As pointed out, the microcalcifications are characterized as to be tiny spots of calcium deposits localized or broadly diffused alone the breast areas or in some cases extremely minute and grouped in clusters. For this particular characterization of such lesions, in our model we enriched the information coming from the texture analysis with the information about points and corners of interest that can be identified in the ROIs. Specifically, they were obtained by using SURF and MinEigenAlg.

**Fig. 2:**
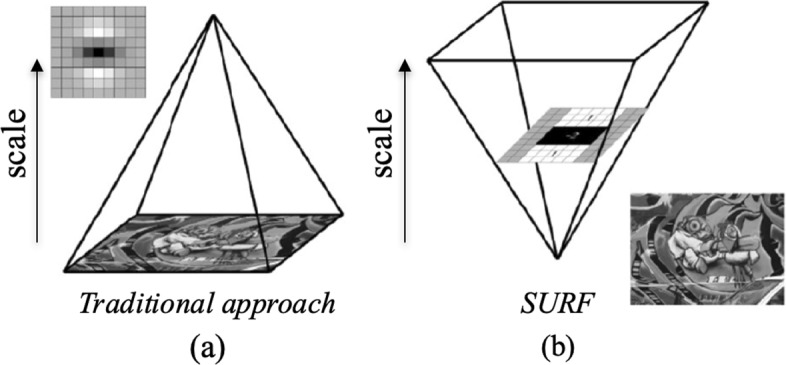
Scale space representation. **a** Traditional approach with a serial downsampling of an image. **b** Surf approach with a parallel upscaling of an image [29]

SURF method [29] is an interest local point detector and descriptor that relies on integral images for image convolutions. It consists of three main parts: interest point detection, local neighborhood description and matching. For the detection of interest points, first it uses square-shaped filters as an approximation of Gaussian smoothing, and then evaluates the sum of the original image within a rectangle using the integral image and requiring evaluations at the four corners of that rectangle. Then, SURF algorithm employs an integer approximation of the determinant of Hessian matrix of an image *I*, defined at the point *p*(*x*,*y*) and scale *σ* as follows:

2 $$ \mathcal{H}(p,\sigma) = \left (\begin{array}{cc} L_{xx}(p,\sigma) & L_{xy}(p,\sigma) \\ L_{yx}(p,\sigma) & L_{yy}(p,\sigma) \end{array} \right),   $$

where *L*_*xx*_(*p*,*σ*) is the convolution of the second-order derivative of Gaussian with the image *I*(*x*,*y*) at the point *x*, and similarly for *L*_*xy*_, *L*_*yx*_ and *L*_*yy*_; the scale *σ* represents the layers obtained by filtering the image with gradually bigger mask (9x9, 15x15, 27x27, etc.). In this way, a pyramidal scale space is built: rather than serial downsampling (Fig. 2a), each successive level of the pyramid is built by upscaling the image in parallel (Fig. 2b) [29]. In order to find points of interest in the image and over scales, local change around the point is measured and detected points are highlighted. Finally, the maxima of the Hessian matrix determinant are interpolated in scale and image space. The descriptor of local neighborhood is made by means a description of the intensity distribution of the pixels within the neighborhood of the point of interest in order to provide a solid description of an image feature. The SURF descriptor fixes a reproducible orientation by using information from a circular region around the points of interest, and adds the Haar wavelet responses. Then, the interest region is split into smaller sub-regions, and for each of them, the Haar wavelet responses are extracted. Finally, the responses are weighted with a Gaussian in order to offer more robustness for deformations, noise and translation. For the last part of the algorithm, matching pairs can be found by comparing the descriptors obtained from different images.

**Fig. 3:**
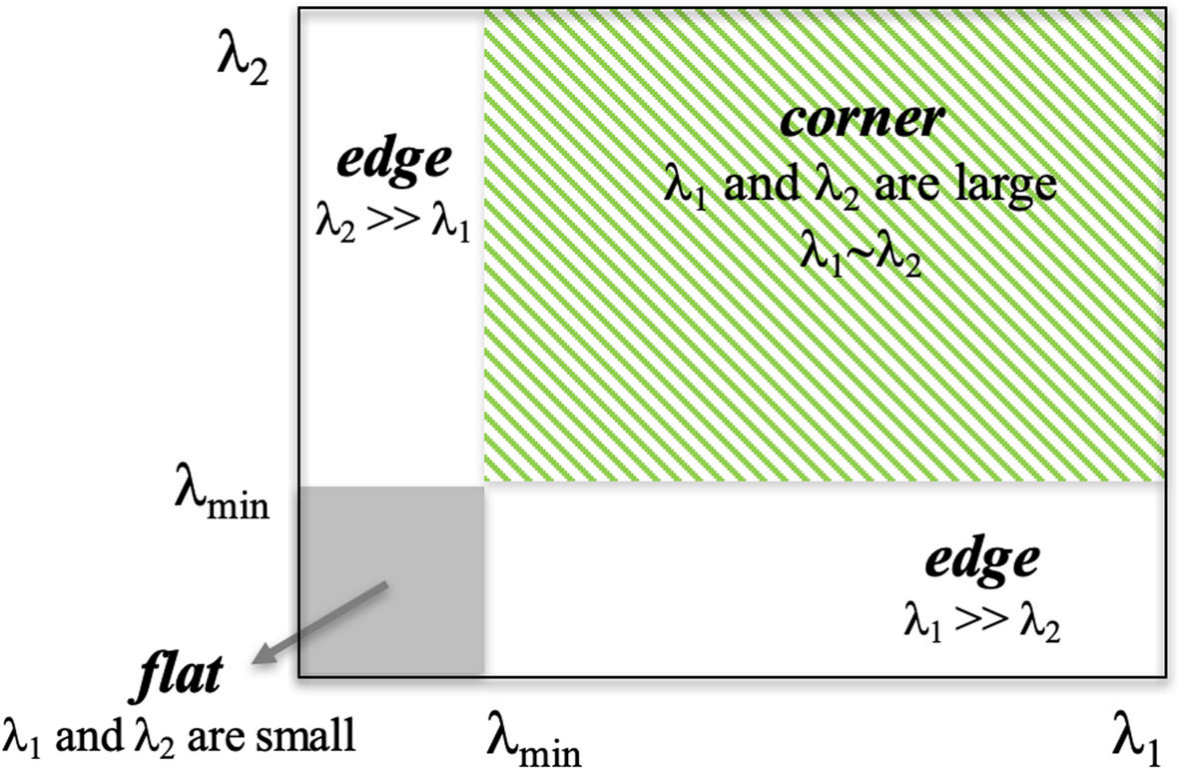
Shi-Tomasi score. In the (*λ*_1_,*λ*_2_) space, only when *λ*_1_ and *λ*_2_ are above a minimum value *λ*_*min*_, the point is considered as a corner (green hatched area). The white and gray areas represent the conditions in which the point is marked as an edge and a flat region, respectively

MinEigenAlg uses the Shi-Tomasi detector to identify the interest corners of an image. It is based on the Harris corner detector [30] with a modification in the score calculation. A corner can be defined as a point where two dominant and different edge directions meet in a local neighborhood of the point, differently from an edge with no change along the edge direction and from a flat region where no change are in all directions. The corner selection criterion of the Harris corner detector is that a score is calculated for each pixel with respect to all the directions (horizontal, vertical and on the two diagonals) by using the two eigenvalues (*λ*_1_ and *λ*_2_) of a symmetric matrix, known as Harris matrix. According to this corner detector, the Harris matrix provides two “large” eigenvalues for an interest corner. Then, a function taking into account the determinant and trace of the Harris matrix gives the following score:

3 $$ R_{H} = \lambda_{1}\lambda_{2} - k (\lambda_{1}+\lambda_{2})^{2},   $$

where *k* is an empirical constant (*k*=0.04−0.06). The Shi-Tomasi corner detector is different from the Harris corner detector in the score computation:

4 $$ R_{ST} = min(\lambda_{1},\lambda_{2}),   $$

where the score depends only on two eigenvalues and not on *k* constant. Considering this minimum value between two eigenvalues as score, when it is greater than a minimum value, the point can be marked as a corner (green hatched area in Fig. 3).

In this preliminary approach, only the number of interest points (IP) and corners (IC) has been taken into account by applying the two algorithms above described.

**Fig. 4:**
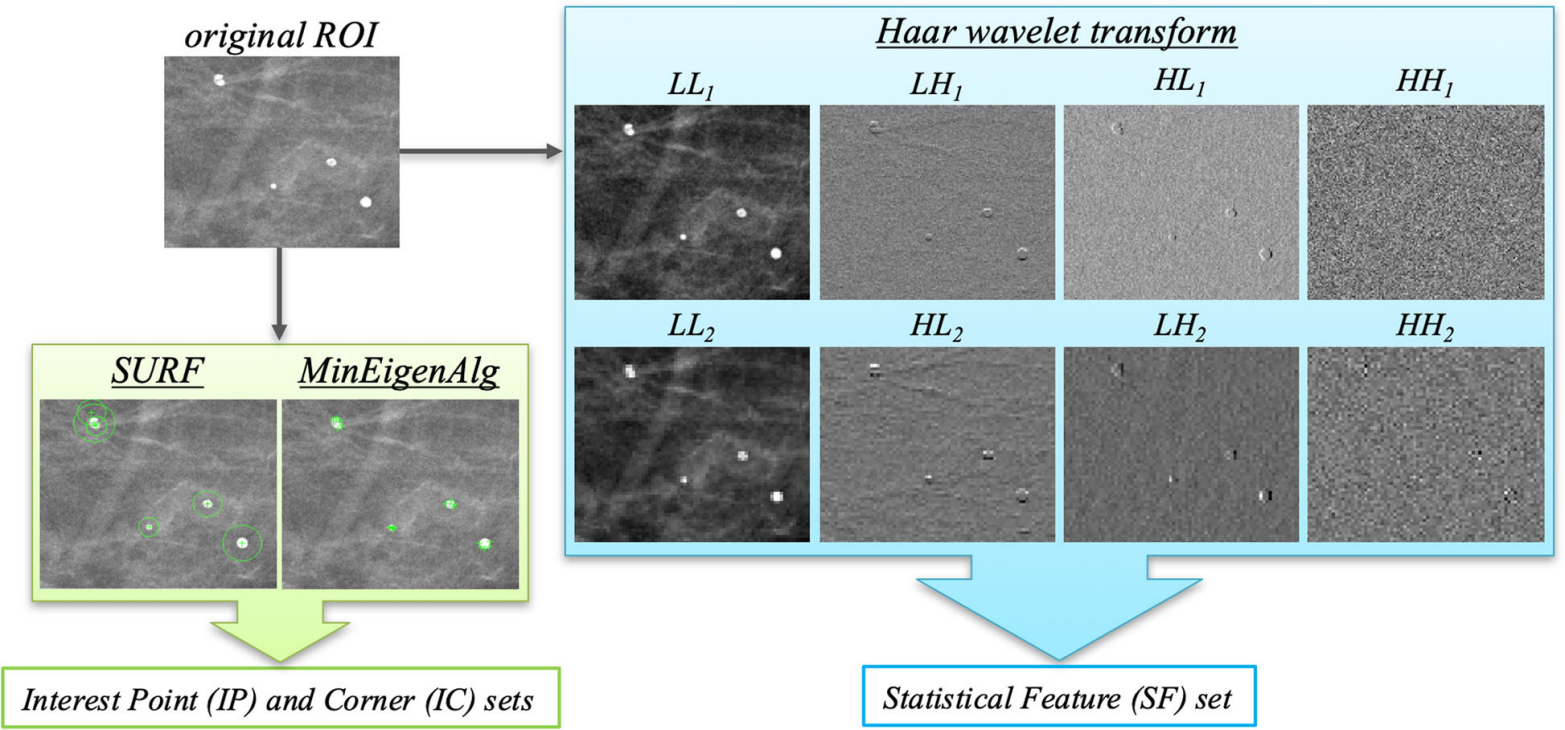
Example of feature set extraction from an original ROI containing microcalcifications. The Statistical Features set is obtained from eight sub-ROIs decomposed by Haar wavelet transform, while the Interest Point and Corner sets are formed by counting the number of points and corners of interest extracted by SURF and MinEigen algorithms, respectively

An example of the feature set extraction is shown in Fig. 4: the SF, IP and IC sets are obtained starting from an original ROI including microcalcifications by applying Haar wavelet transform, SURF and MinEigen algorithms, respectively. The extraction of these feature sets was performed by using the corresponding default functions implemented in the MATLAB R2017a (Mathworks, Inc., Natick, MA, USA) software.

### Classification model

**Fig. 5:**
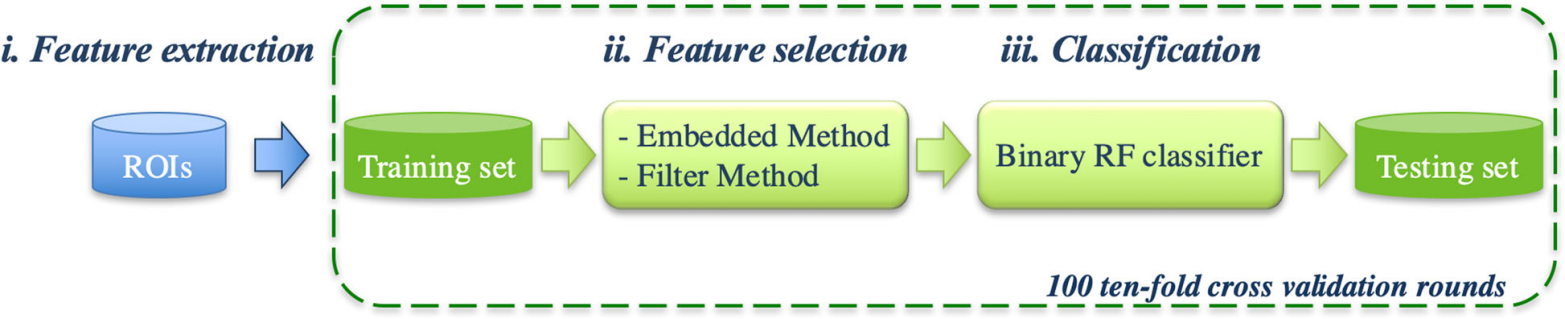
Flow-chart of the proposed model. In a first phase, a set of features on each ROI is extracted, then the feature selection step is performed; finally the RF classifier is trained for the resolution of the binary problem - normal vs abnormal and benign vs malignant

The general structure of the classification model proposed is showed in Fig. 5. The method is developed in three phases: (i) for each ROI a set of features are extracted by using the methods above described; (ii) a features subset is selected on training set; (iii) finally, a Random Forest (RF) binary classifier [33] is trained to discriminate ROIs using the selected features sub-set.

In this work, we evaluate two different kinds of feature selection techniques, such as filter and embedded methods [31, 32]. The filter methods include algorithms that evaluate the capacity of the individual features to predict the expected result. Usually, univariate parametric and non-parametric statistical tests are used to evaluate the significance of the different distributional form of features in sub-samples (classes). Since the feature selection procedure is independent from the machine learning algorithm used, it is possible that the selected features in the first phase will result in a subset that may not work very well downstream of the learning algorithm. Although the learning pipeline is faster, the contribution to discrimination problem generated by the combination of features is lost. In our work, in order to filter out features that have little chance to be useful in data analysis, we used the non-parametric Wilcoxon-Mann-Whitney test [36] to verify whether the medians of distributions of the two classes of the binary problem are equal.Embedded methods allow optimization between the interaction of the selected features and the classification algorithm used. In fact, the selection criterion is grafted into the chosen machine learning algorithm: an analysis of feature importance with respect to its expected result is intrinsically elaborated, therefore these methods are essentially fulfilling the goal, i.e. optimizing the classifier performance. However, they are computationally more expensive than the repeated learning steps and cross-validation. In this work, the feature relevance problem-driven is calculated by the same RF [33] that is often used for feature selection task. Indeed, the tree-based strategy used by random forests naturally ranks by how well they improve the purity of the node: nodes with the greatest decrease in impurity happen at the start of the trees, while nodes with the least decrease in impurity occur at the end of trees. Thus, by pruning trees below a particular node, we can create a sub-set of the most important features.

At each step of cross-validation, a feature ranking is calculated with respect to their predictive power assessed with the two different approaches (filter and embedded method) on training set. Then, a binary classification model is trained by selecting iteratively an increasing number of features sorted by their discriminating power.

By using the filter method, the features are sorted in descending order by the p-value non-parametric test calculated on the training dataset; on the contrary, with the embedded method the features are sorted in increasing order by the relevance calculated on the training set. The subset selected at each step of the cross-validation is in turn used to train a RF classifier: a standard configuration was adopted with 100 trees and 20 features (as described in [33]) randomly selected at each split.

The proposed model was evaluated on two binary discrimination problems, i.e. normal vs abnormal tissue and benign vs malignant lesions. The performance of the prediction models was evaluated in terms of Area Under the Curve (AUC) of the Receiver Operating Characteristic (ROC) curve, accuracy, sensitivity and specificity on 100 ten-fold cross-validation rounds.

## Results

In a previous work [37], we proposed a CAD for characterizing and discriminating ROIs that did not provide a real feature selection process. It was trained on statistical features calculated on the multiscale decomposition of the image based on the Haar wavelet transform, and on interest points and corners detected by using two known algorithms, SURF and MinEigenAlg. In particular, a state-of-the-art machine learning classifier, such as a Random Forest classifier, was trained to solve a binary discrimination problem. The performance of the proposed model was evaluated in cross validation on 260 ROIs (130 normal, 75 benign and 55 malignant ROIs); the experimental outcomes showed that the developed model was high performing both for the normal vs abnormal classification problem, with a median AUC value of 98.46*%* and an accuracy of 95.83*%*, and for benign vs malignant one, with a median AUC value of 94.19*%* and an accuracy of 88.19*%*.

Starting from these encouraging results, here we would examine the contribution of different methods on the feature selection process, and consequently on the learning performance, as well as analyze the discrimination power of some features according to their assigned relevance on a larger and different set of ROIs.

Specifically, as previously reported, two different approaches for the feature selection task were evaluated, namely filter and embedded methods and the performance of the classification model in cross-validation was measured. In particular, as described in the “Materials and methods” section, at each step of cross-validation, a features ranking is calculated with the two approaches on training set and a binary RF classifier is trained by selecting iteratively an increasing number of features.

**Fig. 6:**
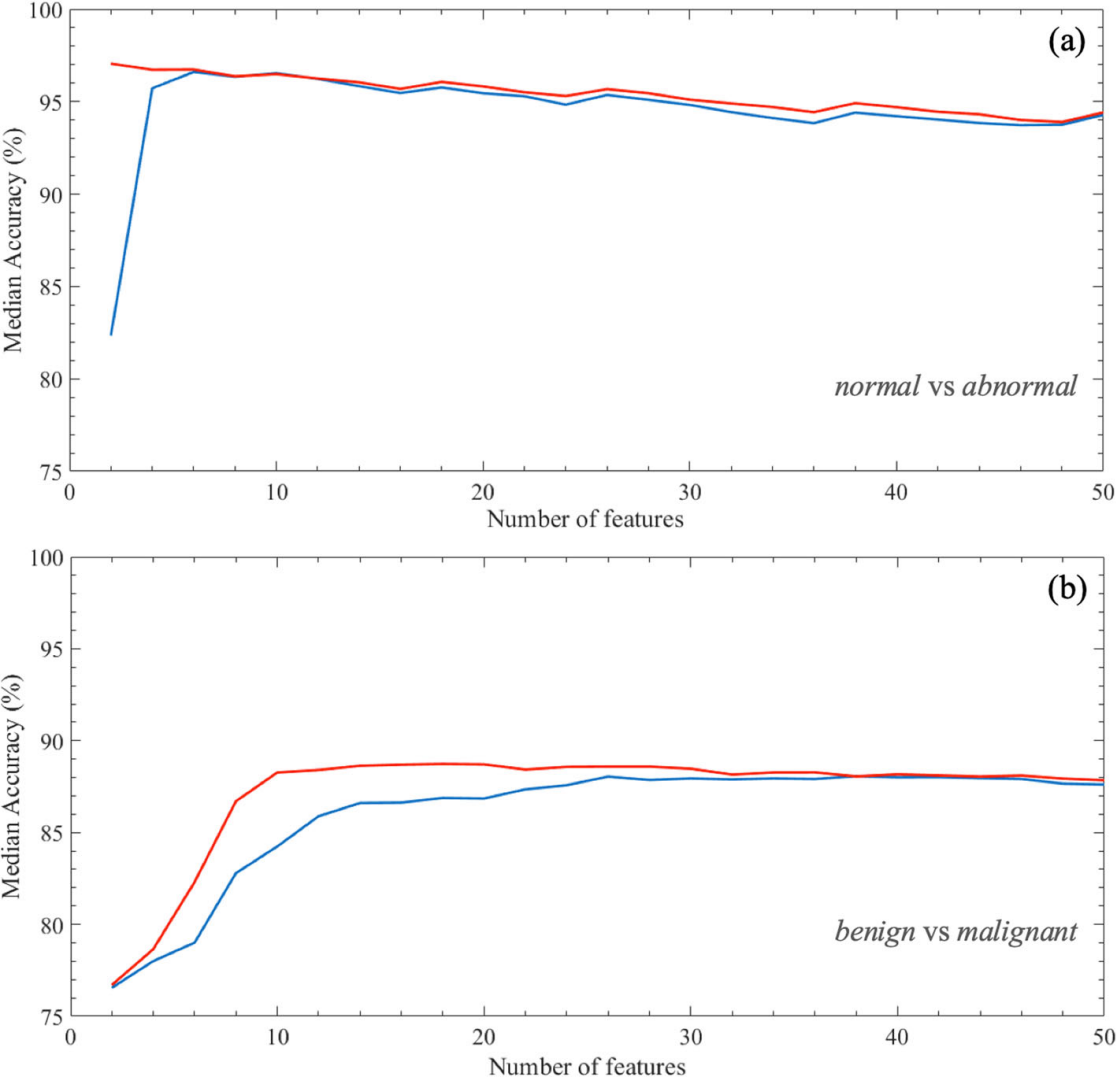
Median accuracy (%) with the growth of the number of features. The median value is calculated on 100 rounds of 10-fold cross validation for increasing values of the number of features used to train the proposed model to classify ROIs into **a** normal/abnormal **b** and benign/malignant. Two different feature selection approaches are used, that are embedded (red line) and filter (blue line) methods

Figure 6 shows the performance results for solving the normal/abnormal (a) and benign/malignant (b) problems on ROIs extracted from BCDR dataset. The mean accuracy of classifier models (%) has been calculated on 100 rounds of 10-fold cross validation. The experiment results show that the number of features needed to optimize the performance for discriminating normal and abnormal ROIs are 2 in case of the embedded method is used, whereas they become 6 with the exploitation of the filter method. In particular, the embedded method always selects only the interest points and corners with SURF and MinEigen algorithms, respectively; instead, among the first 6 most significant features selected with the filter method there is also the kurtosis measurement calculated on different Haar decompositions (Table 1).

**Table 1:** Significant features on BCDR database

Normal/Abnormal				Benign/Malignant			
Embedded M.	freq (%)	Filter M.	freq (%)	Embedded M.	freq (%)	Filter M.	freq (%)
(k ≤2)		(k ≤6)		(k ≤10)		(k ≤26)	
# Interest Points	100	# Interest Points	100	Variance _LL2	100	Variance _LL1	100
# Interest Corners	100	Kurtosis _HL2	99.80	# Interest Corners	100	Skewness _LL1	100
		# Interest Corners	99.10	Variance _LL1	99.90	Entropy _LL1	100
		Kurtosis _HL1	97.80	RelSmoothness _LL2	99.90	RelSmoothness _LL1	100
		Kurtosis _LH1	76.40	RelSmoothness _LL1	99.60	Entropy _HL1	100
		Kurtosis _LH2	61.90	# Interest Points	91.30	Entropy _HH1	100
		Variance _LH2	24.80	Variance _HH1	77.70	Kurtosis _HH1	100
		RelSmoothness _LH2	21.90	RelSmoothness _HH1	77.40	Variance _LL2	100
				Entropy _HH1	58.90	Skewness _LL2	100
				Entropy _HL1	44.80	Entropy _LL2	100
				Mean _HH1	41.20	RelSmoothness _LL2	100
						Kurtosis _LH2	100
						Kurtosis _HL2	100
						Kurtosis _HH2	100
						# Interest Points	100
						# Interest Corners	100
						Entropy _LH1	99.20
						Entropy _LH2	98.60
						Entropy _HH2	97.80
						Kurtosis _HL1	97.10
						RelSmoothness _HH1	96.10
						Variance _HH1	88.80
						Skewness _HL2	76.30
						Mean _LL1	59.00

The features whose occurrence in the first k positions of the rankings defined by the filter and embedded methods is significantly different from the case (p-value null model test ≤0.05) are reported. k is the number of features that maximizes the accuracy of normal vs abnormal and benign vs malignant classification problems

The discrimination problem of the benign and malignant ROIs requires more features to solve the classification problem; specifically, 10 features with the embedded method and 26 with the filter method. Among the first 10 most significant features selected with the first method, there are yet interest points and corners provided by SURF and MinEigen algorithms, respectively, but also relative smoothness, variance and entropy measurements calculated on different Haar decompositions. With reference to the features selected by the filter method, skewness and kurtosis measurements result also significant.

Table 2 shows the best classification performances calculated on 100 rounds of 10-fold cross-validation. The binary models trained to discriminate normal/ abnormal ROIs by using the two different feature selection approaches are highly performing; however, the performance of the model trained on features selected by the embedded method is slightly higher (p-value Wilcoxon-Mann Whitney test ≤0.01) of those obtained with the filter approach, except in the identification of malignant ROIs (sensitivity); in particular, with only 2 features, it reaches a median AUC value of 98.16*%*, an accuracy of 97.31*%*, a sensitivity of 94.62*%*, and a specificity of 100%.

**Table 2:** Best classification performance on BCDR database

		Normal/Abnormal	Benign/Malignant
**Embedded** **Method**	AUC	98.16 (97.87−98.48)∗∗	92.08 (91.61−92.58)
	Accuracy	97.31 (96.92−97.31)∗∗	88.46 (87.69−89.23)
	Sensitivity	94.62 (93.85−94.62)	89.09 (87.27−90.91)
	Specificity	100 (100−100)∗∗	88.00 (86.67−89.33)
**Filter** **Method**	AUC	98.67 (98.57−98.76)	92.13 (91.66−92.78)
	Accuracy	96.92 (96.54−96.92)	87.69 (86.92−89.23)
	Sensitivity	93.85 (93.85−94.62)	89.09 (87.27−90.91)
	Specificity	99.23 (99.23−100)	87.33 (85.33−89.33)

The classification performance calculated in correspondence with the best result highlighted in the 100 rounds of 10-fold cross-validation for increasing the number of selected features, are summarized. We tested the significance of the diversity of performance measures obtained with the two different feature selection techniques on the same classification problem. Statistical significance is measured with the Wilcoxon-Mann-Whitney test: ** p-value <0.01 (Bonferroni correction)

Indeed, the models trained to discriminate benign/malignant ROIs with two different feature selection methods are yet highly performing and significantly comparable (p-value Wilcoxon-Mann Whitney test ≤0.01), however the embedded method selected fewer features: with 10 features selected by the embedded feature selection method, the classification performances reaches a median AUC value of 92.08*%*, an accuracy of 88.46*%*, a sensitivity of 89.09*%*, and a specificity of 88.00*%*.

## Discussion

In this work we have developed a binary classification model of ROIs containing microcalcification clusters. Firstly, for each ROI obtained from images of BCDR database, we have extracted textural features on a multiscale decomposition based on the Haar wavelet transform, and also detected interest points and corners by using two known algorithms, SURF and MinEigenAlg. In particular, we have evaluated the classification performance of a RF classifier for increasing values of features selected on training set of the cross-validation selected by two different approaches, that are embedded and filter methods.

The experimental results on the dataset considered have highlighted, regardless of the method of feature selection used, the normal/abnormal problem can be effectively solved with a number of features decidedly contained (no more than 6), achieving high performance comparable to the state-of-the-art. Specifically, the best performance is obtained with the embedded feature selection method using only two features, that are interest points and corners provided by SURF and MinEigenAlg, respectively.

For what concerns the much more complex problem of benign/malignant classification, which represents the main focus of the works proposed in the literature about the characterization of microcalcification clusters, the best results of the proposed method (median AUC value of 92.08*%*, accuracy of 88.46*%*) were obtained using 10 features selected by the embedded method: the joint contribution of these has allowed the achievement of comparable performance with respect to the best result obtained by independently selecting the 26 features with the highest discriminated power.

Experimental results showed that interest points and corners, relative smoothness, variance and entropy measurements calculated on different Haar decompositions seem to have significant information content for discriminating benign/malignant ROIs.

Results of this study are quite promising. Table 3 shows the performance of state-of-the-art models for the classification into benign and malignant microcalcifications. For this comparison, works on classification of microcalcification clusters mainly using textural features [38] or a combination of these with other types of features [21], but also topological ones [17, 25] and statistical features [19] were taken into account. Moreover, different machine learning approaches and databases have been used. Therefore, the comparison is purely qualitative. However, the classification performances obtained by our approach are more performing with respect to these works and do not require a manual segmentation of the lesion by radiologist but only the identification of a suspicious area. Moreover, the proposed approach is able to characterize the lesions by exploiting a reduced set of features.

**Table 3:** Benign vs Malignant microcalcifications: accuracy (Acc) and Area Under the Curve (AUC) performances

Method	No. ROIs	Feature type	Classifier	Acc (%)	AUC (%)
Chen et al. (2015) [17]	300	topological features	kNN	85	91
Ren et al. (2012) [19]	295	statistical features	kNN	82	86
Khehra et al. (2013) [21]	380	statistical, shape and	LS-SVM	89	89
		textural features			
Strange et al. (2014) [25]	300	mereotopological features	Barcodes	80	82
Hu et al. (2017) [38]	150	textural features	ELM	-	92
**Proposed** **approach**	**260**	**textural** **features** **and**	**RF**	**88**	**92**
		**#interest** **points/corners**			

In the next stage of our studies, we will evaluate the proposed model on different databases and also evaluate other features to improve the classification performances of benign and malignant microcalcification clusters.

## Conclusion

The diagnosis of microcalcifications is usually based on radiologists expertise sometimes resulting in an inaccurate lesion detection with unnecessary biopsies and subsequent surgery.

Several methods have been developed for the task of microcalcification diagnosis, in some cases well performing. Nevertheless, the automatic and accurate classification of microcalcification clusters, especially in differentiating the benign from the malignant ones, remains still complicated due to their nature. In this paper, we propose the use of texture analysis methods combined with machine learning techniques in order to select the optimal subset for characterizing breast regions. In particular, we trained a binary RF classifier on an increasing number of features sorted by their statistical significance in the set of data; these features were calculated using two different feature selection approaches, such as embedded and filter methods.

Both feature selection techniques revealed highly performing, nevertheless the best accuracy result obtained with embedded method is more parsimonious than the filter one: it needs only 2 features to discriminate ROIs into normal/abnormal and 10 into benign/malignant.

The measurements provided by SURF and MinEigen algorithms seems to provide a strong informative content useful for the characterization of microcalcification clusters. In this work we have limited ourselves to considering only the number of interest points and corners and not also the features associated with them. In order to improve the performances, specially in the classification of benign and malignant microcalcifications, it would be interesting to deepen the analysis of these features. Future work will concern also the simultaneous exploitation of the information coming from the CC and MLO views of the same breast lesion [39] and the evaluation of more complex features selection methods by wrapped approach.

## Data Availability

The images and data used in this analysis are extracted from the public database Breast Cancer Digital Repository (BCDR) available on https://bcdr.ceta-ciemas.eslink.

## Abbreviations

ANN: Artificial neural networks
AUC: Area under the curve
BCDR: Breast cancer digital repository
CAD: Computer-aided detection/diagnosis
CC: CranioCaudal
DM: Digital mammography
FM: Film mammography
HH: High-high
HL: High-low
IC: Interest corner
IP: Interest point
kNN: k-nearest neighbours
LH: Low-high
LL: Low-low
MC: MicroCalcification
MinEigen(Alg): Minimum eigenvalue (algorithm)
MLO: MedioLateral oblique
RF: Random forest
ROC: Receiver operating characteristics
ROI: Region of interest
SF: Statistical feature
SURF: Speed up robust feature
SVM: Support vector machine
